# Response of Intestinal Microbiota of Tiger Puffer (*Takifugu rubripes*) to the Fish Oil Finishing Strategy

**DOI:** 10.3390/microorganisms11010208

**Published:** 2023-01-13

**Authors:** Yaoyao Kong, Zhangbin Liao, Xiuhua Ma, Mengqing Liang, Houguo Xu, Kangsen Mai, Yanjiao Zhang

**Affiliations:** 1 Key Laboratory of Aquaculture Nutrition and Feed, Ministry of Agriculture, The Key Laboratory of Mariculture, Ministry of Education, Ocean University of China, 5 Yushan Road, Qingdao 266003, China; 2 Yellow Sea Fisheries Research Institute, Chinese Academy of Fishery Sciences, 106 Nanjing Road, Qingdao 266071, China; 3 Qingdao National Laboratory for Marine Science and Technology, 1 Wenhai Road, Qingdao 266237, China

**Keywords:** fish oil replacement, lipid source, intestinal health, intestinal microbiota, *Takifugu rubripes*

## Abstract

The fish oil finishing (FOF) strategy, that is, re-feeding fish with fish oil (FO)-based diet after a certain period of feeding with alternative lipid source-based diets. On tiger puffer, the present study investigated the response of intestinal microbiota to FOF. Fish were fed four diets based on FO, soybean oil, palm oil and beef tallow as lipid sources, respectively, firstly for 50 days (growing-out period), and then fed the FO-based diet for 30 more days (FOF period). The results showed that dietary terrestrially sourced oils impaired the intestinal function in the growing-out period. However, the activities of amylase, trypsin and anti-oxidative enzymes (SOD, CAT, T-AOC), as well as gene expression of inflammatory cytokines (IL-1β, TNF-α, TGF-β) and tight junction protein (Claudin4, Claudin7, Claudin18, JAM, ZO-1) in the intestine were significantly recovered by FOF. The 16S rDNA sequencing analysis showed that FOF improved the similarity of bacterial community among the groups. The MetaStat analysis confirmed that FOF regulated the abundance of butyric acid-producing bacteria (*Lachnospiraceae*, *Eubacterium*, *Butyricicoccus*, *Clostridium* and *Roseburia*) and bacteria related to digestion and absorption (*Sphingomonas*, *Romboutsia* and *Brevibacillus*). In conclusion, FOF can recover the intestine function. The intestinal microbiota probably participated in and played a key role in the recovery process.

## 1. Introduction

In the period 1990–2020, total world aquaculture expanded by 609% in annual output with an average growth rate of 6.7% per year, and aquatic food, as the third largest source of human food protein in the world, provided about 17% of animal protein and 7% all protein in 2019 [1]. With the rapid development of aquaculture industry, fish oil (FO) is in relative short supply and the price is increasing, making the FO replacement by terrestrially sourced oils (TSO) essential [2,3,4,5,6,7]. However, TSO have been reported to have negative impact on the fish growth performance, immunity, intestinal health and in particular muscle quality, as observed in species such as Atlantic salmon (*Salmo salar*), red claw crayfish (*Cherax quadricarinatus*), Nile tilapia (*Oreochromis niloticus*), large yellow croaker (*Larimichthys crocea*), golden pompano (*Trachinotus ovatus*), gilthead bream (*Sparus aurata*), and rainbow trout (*Oncorhynchus mykiss*) [8,9,10,11,12,13]. Various measures have been applied to mitigate these negative effects [14,15,16], among which FOF has been demonstrated to be able to improve the growth performance and muscle fatty acid composition, as observed in Florida pompano (*Trachinotus carolinus*), Atlantic salmon and tiger puffer (*Takifugu rubripes*) [17,18,19].

The intestine is the main site for fish to digest and absorb nutrients. It also participates in a variety of stress responding processes. Therefore, the destruction of intestinal health will cause disorder of nutrient metabolism and disease resistance [20,21]. Intestinal microbiota has been a focus due to its key roles in maintaining intestinal health [22]. In addition, the intestinal microflora also plays a crucial role in nutrients digestion and absorption of host gut, and secrete and metabolize various short-chain fatty acids [23,24,25]. The composition of intestinal microbes is significantly affected by diets. However, there has been no study reporting the intestinal microbiota in response to FOF. Tiger puffer is a fish belonging to the genus *Takifugu* in the family Tetraodontidae of the order Tetraodontiformes. It has the highest economic value in the genus *Takifugu*. As a species that can be reared in both factory and sea cages, the total production of tiger puffer in China in increasing and the relevant production of its compound feed is increasing [26]. In addition, because of the complete sequencing of the genome, tiger puffer is also often used as a model fish for scientific research. This study aims to evaluate the response process of tiger puffer intestinal microbiota under FOF, and to explore its specific role in the overall recovery process of host. These results will help to elucidate the mechanisms involved in the changes of fish intestinal microbiota during diet shift.

## 2. Materials and Methods

### 2.1. Ethics Statement

All experimental protocols involved in this study were approved by the Animal Care and Use Committee of Yellow Sea Fisheries Research Institute on 12 October 2019 (recorded case No.: IACUC201910123627).

### 2.2. Diets

Four isonitrogenous and isolipidic experimental diets were prepared with fish oil (FO), soybean oil (SO), palm oil (PO) and beef tallow (BT) as main lipid source, respectively (Table 1). Feed production and storage operations were carried out in accordance with our laboratory standards [27].

### 2.3. Feeding Trial

Juvenile tiger puffer (19.50 ± 0.01 g) was purchased from Tangshan Haidu Aquatic Food Co., Ltd., (Tangshan, China). The fish were fed with the control diet for a week to acclimate to the experimental conditions. At the end of acclimation, healthy fish were weighed and randomly allocated to 12 cages, with three replicate cages per treatment. Each cage was stocked with 50 fish. Fish were hand-fed to apparent satiation twice daily (6:00 and 18:00). The four experiment diets were assigned to each group during the first 50 days (the growing-out period), and then the FO control diet was fed to all groups for 30 more days (the FOF period). During the feeding period, the experimental conditions were as follows: water temperature: 20–23 °C, dissolved oxygen: 5–7 mg/L, salinity: 22–30, pH: 7.4–8.2. The specific feeding process was described in a previous article [18].

### 2.4. Sample Collection

Sampling was conducted at the end of the both growing-out and FOF periods. For each treatment, 27 fish were randomly collected for gut sampling. The scissors and tweezers were disinfected with 70% alcohol before sampling, and the whole operation was carried out under the alcohol lamp to ensure a sterile environment. The hindgut was divided into three equal sections with sterilized scissors and forceps, and the intestinal mucosa was scraped off, then quickly frozen and stored at −80 °C.

### 2.5. RNA Extraction, cDNA Synthesis and Quantitative Real-Time PCR

RNAex Pro Reagent (AG21102, Accurate Biotechnology (Hunan) Co., Ltd., Changsha, China) was used to extract the total RNA from the hindgut samples. RNA quality was assessed by agarose electrophoresis and Nano Drop^®^ ND-1000 according to the following criteria: (1) the electrophoretic map shows three clear and complete bands; (2) the 260/280 ratio of RNA is 1.8–2.0; (3) the 260/230 ratio is 2.0–2.2, the extracted RNA is of high quality and can be used for subsequent experiments. The RNA samples extracted above were synthesized into cDNA by Eppendorf Mastercycler gradient PCR (Eppendorf, Germany) and Takara’s reverse transcription kit, and the obtained cDNA products were stored at −20 °C.

In the PCR study, both the internal reference gene (β-actin) and the target gene sequences were obtained from the NCBI database; the primer was designed on the NCBI; and the final products were synthesized by Sangon Biotech (Shanghai, China) Co., Ltd. The primer information is shown in Table 2.

The kit used for real-time fluorescence quantitative PCR is SYBR^®^ Green Premix Pro Taq HS qPCR Kit (AG11701, Accurate Biotechnology (Hunan) Co., Ltd., Changsha, China). Reaction system: 2 × SYBR Green Real-time PCR Master Mix, 12.5 μL; cDNA, 1 μL; Primer (10 μM), 1 μL; dH_2_O, 10.5 μL. Reaction process: 95 °C for 2 min; 95 °C for 10 s; 60 °C for 10 s; 72 °C for 20 s, 39 cycles repeated from the second step. Analysis of dissolution curve: from 58 to 95 °C; increasing by 0.5 °C; 5 s each grade. The expression levels of these genes were calculated according to the 2^−ΔΔCT^ method [28].

### 2.6. Determination of Intestinal Enzyme Activity

The activities of enzymes related to immunity, antioxidation and digestion in the intestine of tiger puffer were measured: amylase, trypsin, alkaline phosphatase (AKP), acid phosphatase (ACP), catalase (CAT), superoxide dismutase (SOD) and total antioxidant capacity (T-AOC). The content of malondialdehyde (MDA) in the gut was also measured. The intestinal samples were thawed at 4 °C and then diluted with saline according to the mass ratio. All commercial kits were supplied by Nanjing Jiancheng Bioengineering Research Institute, and the specific steps are slightly modified according to the actual situation.

### 2.7. DNA Extraction and High Throughput Sequencing of Intestinal Microbiota

The kit used for genomic DNA extraction of intestinal microbiota was QIAamp PowerFecal Pro DNA Kit, and the operation steps were carried out according to the instructions of the kit. The intestinal mucosa was fully mixed with 800 μL CD1, and then the supernatant was mixed with 200 μL CD2 after centrifugation. After centrifugation, the supernatant was mixed with 600 μL CD3 and the same volume of lysate was added. After centrifugation, the filtrate was discarded and the previous steps were repeated. Then, 500 μL EA was added, and after centrifugation, the filtrate was discarded and 500 μL C5 was added. After centrifugation, the filtrate was discarded and 60 μL C6 was added. Finally, after centrifugation the DNA product was obtained after the MB column was discarded.

To analyze the differences in the gut microbial community among different treatment groups, the V4 region of 16S rDNA of 24 samples in the intestine was amplified. The obtained product was run on agarose gel electrophoresis and the samples with a brightness of 400–450 BP in the main band were selected for subsequent experimental operation. The TruSeq DNA PCR-Free Library Preparation Kit was used to build the library, and the sequencing library was generated according to the steps in the manual. After the quality evaluation, the constructed library was sequenced according to the PE 250 strategy and selected on the Illumina novaseq platform (Tianjin Novogene Genomics Technology Co. Ltd., Tianjin, China).

### 2.8. Sequencing Data Analysis of Intestinal Microbiota

The intestinal sample information of each processing group is separated from the off-line data. After the linker sequence information was deleted, the reads of each sample were spliced into Raw Tags by Flash software [29]. The new sequence was filtered and analyzed through a series of strict and complex operations to generate Clean Tags [30]. At this time, the existing sequences were also doped with chimeric sequences, which were compared with the annotation library and eliminated to obtain Effective Tags [31]. Uparse software (Uparse v7.0.1001) was used to cluster all Effective Tags of all samples [32]. According to 97% concordance, the sequences were classified as OTU, and the sequence with the highest frequency was used as the representative sequence. Based on the Mothur method, the OTUs sequences were annotated by species through the SSUrRNA database, and the species composition at each taxonomic level was analyzed [33]. The phylogenetic relationship of all OTU representative sequences was compared using MUSCLE software. Then, the least amount of data in the sample was taken as the standard, and the data of all samples were homogenized accordingly [34]. The following diversity analysis used the homogenized data. The MetaStat analysis used R software to determine the bacterial taxa representing the differences between different groups at each classification level [35].

### 2.9. Statistical Analysis

All data except the microbiota sequencing data were tested for normality and variance homogeneity using the Shapiro-Wilk W goodness of fit test and the Bartlett test, respectively. SPSS 22.0 was used to perform one-way analysis of variance on the data, and the mean value among each group was obtained by Tukey’s test. The data were presented as means ± standard error. *p* < 0.05 was regarded as statistically significant.

## 3. Results

### 3.1. Effects of TSO on Nutrient Digestion and Absorption, as well as Anti-Oxidative Capacity in the Intestine at the End of Growing-Out Period

**Figure 1:**
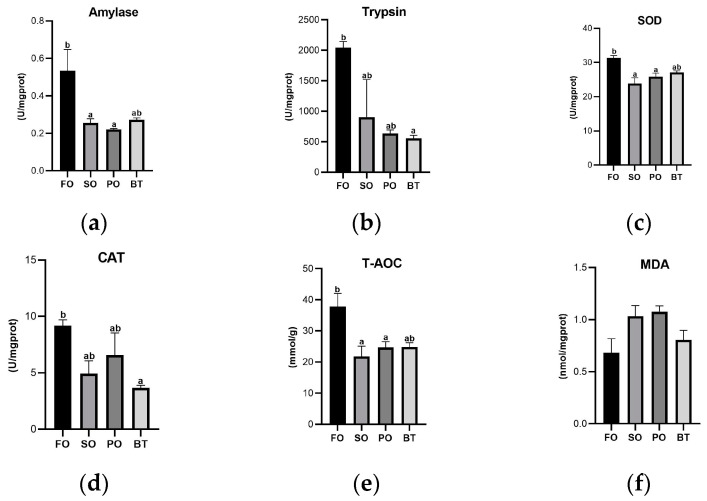
Effects of different diets on intestinal enzyme activities and MDA content of tiger puffer at the end of growing-out period. (**a**) Amylase activity; (**b**) Trypsin activity; (**c**) SOD activity; (**d**) CAT activity; (**e**) T-AOC activity; (**f**) MDA content. Values were means of triplicate tanks with standard errors. ^a, b^ For a certain parameter, value bars sharing a same superscript letter or those without superscript letter are not significantly different as evaluated by Tukey’s test (*p* < 0.05).

At the end of the growing-out period, compared with the FO control group, the activity of amylase significantly decreased in the SO and PO groups (*p* < 0.05) (Figure 1a). The trypsin activity decreased significantly in the BT group (*p* < 0.05) (Figure 1b). The activities of SOD and T-AOC decreased significantly in the SO and PO groups (*p* < 0.05) and the CAT activity decreased significantly in the BT group (*p* < 0.05). There was no significant difference in MDA content between the TSO groups and the FO group (Figure 1c–f).

### 3.2. Effect of TSO on the Intestinal Barrier Function at the End of Growing-Out Period

**Figure 2:**
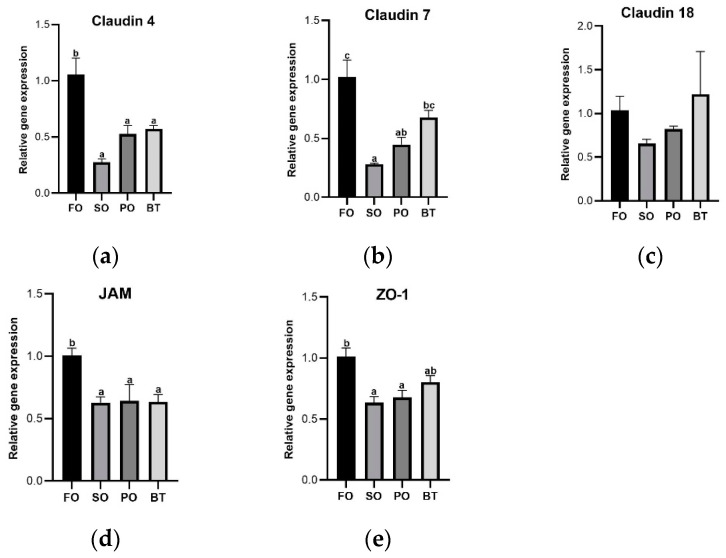
Effects of different diets on the expression of tight junction protein mRNA in the gut of tiger puffer at the end of growing-out period. (**a**) Expression of Claudin 4; (**b**) Expression of Claudin 7; (**c**) Expression of Claudin 18; (**d**) Expression of JAM; (**e**) Expression of ZO-1. Values were means of triplicate tanks with standard errors. ^a, b, c^ For a certain parameter, value bars sharing a same superscript letter or those without superscript letter are not significantly different as evaluated by Tukey’s test (*p* < 0.05).

Compared with FO group, the expression of intestinal mechanical barrier proteins, Claudin 4 and JAM-A, was significantly down-regulated in the TSO groups (*p* < 0.05), and that of Claudin 7 and ZO-1 was also down-regulated in the TSO groups (Figure 2).

**Figure 3:**
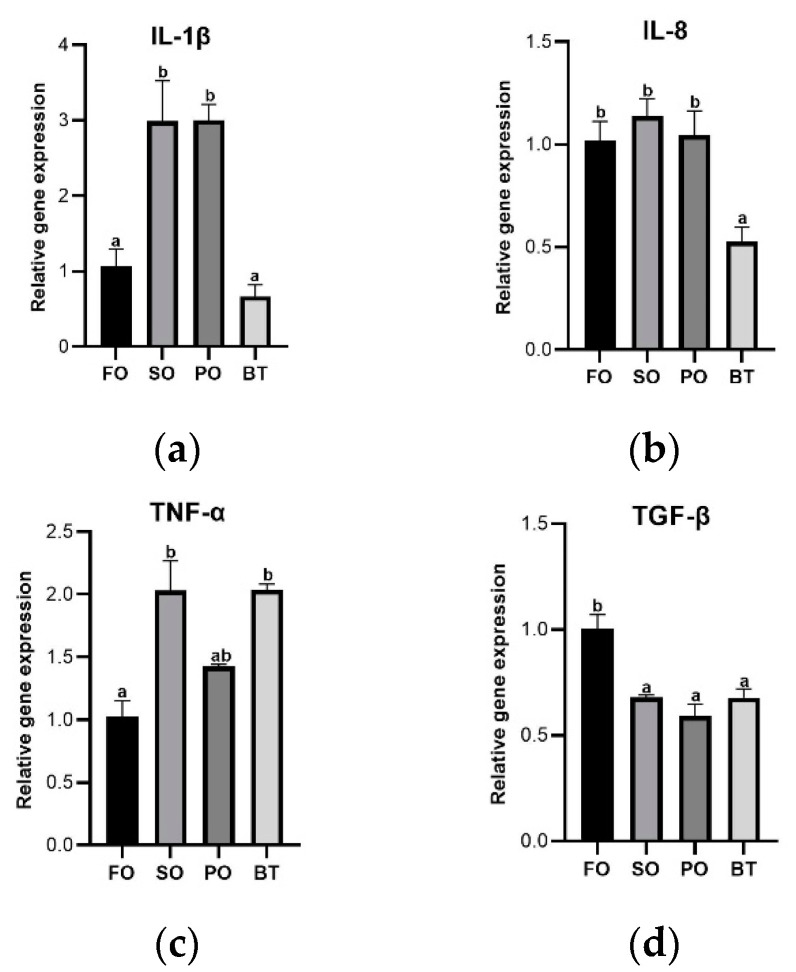
Effects of different diets on the expression of intestinal inflammatory factors of tiger puffer at the end of growing-out period. (**a**) Expression of IL-1β; (**b**) Expression of IL-8; (**c**) Expression of TNF-α; (**d**) Expression of TGF-β. Values were means of triplicate tanks with standard errors. ^a, b^ For a certain parameter, value bars sharing a same superscript letter or those without superscript letter are not significantly different as evaluated by Tukey’s test (*p* < 0.05).

Compared with the FO control group, the gene expression of pro-inflammatory factor IL-1β was significantly up-regulated in the SO and PO groups, and that of TNF-α was significantly up-regulated in the SO and BT groups. The gene mRNA expression of TGF-β was significantly decreased in TSO group (*p* < 0.05) (Figure 3).

### 3.3. Effects of TSO on Nutrient Digestion and Absorption, Antioxidative Capacity and Barrier Function of the Intestine at the End of FOF Period

**Figure 4:**
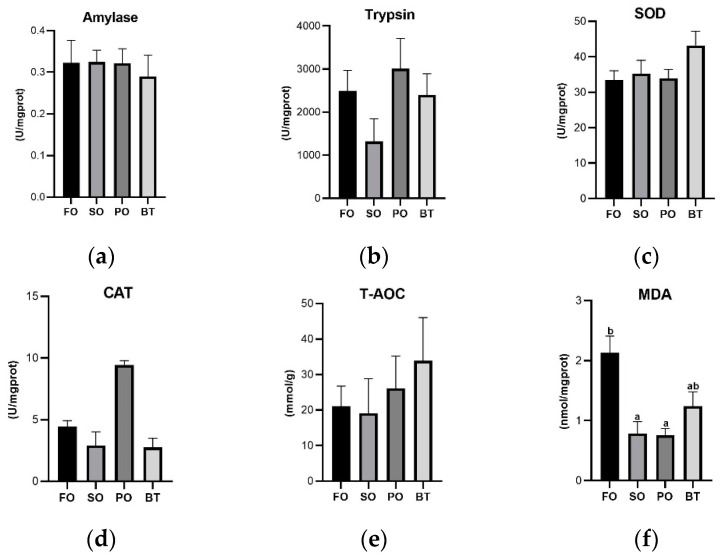
Effects of different diets on intestinal enzyme activities and MDA content of tiger puffer at the end of FOF period. (**a**) Amylase activity; (**b**) Trypsin activity; (**c**) SOD activity; (**d**) CAT activity; (**e**) T-AOC activity; (**f**) MDA content. Values were means of triplicate tanks with standard errors. ^a, b^ For a certain parameter, value bars sharing a same superscript letter or those without superscript letter are not significantly different as evaluated by Tukey’s test (*p* < 0.05).

At the end of the FOF period, compared with the FO control group, there was no significant change in the activities of amylase, trypsin, SOD, CAT and T-AOC in the TSO treatments. Compared to the FO group, the MDA content was significantly lower in the SO and PO groups (Figure 4).

**Figure 5:**
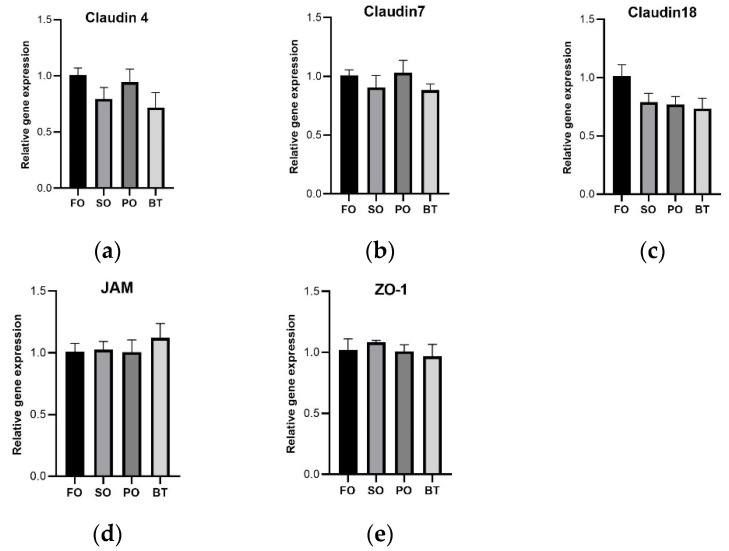
Effects of different diets on the expression of tight junction protein mRNA in the gut of tiger puffer at the end of FOF period. (**a**) Expression of Claudin 4; (**b**) Expression of Claudin 7; (**c**) Expression of Claudin 18; (**d**) Expression of JAM; (**e**) Expression of ZO-1. Values were means of triplicate tanks with standard errors. ^a, b^ For a certain parameter, value bars sharing a same superscript letter or those without superscript letter are not significantly different as evaluated by Tukey’s test (*p* < 0.05).

**Figure 6:**
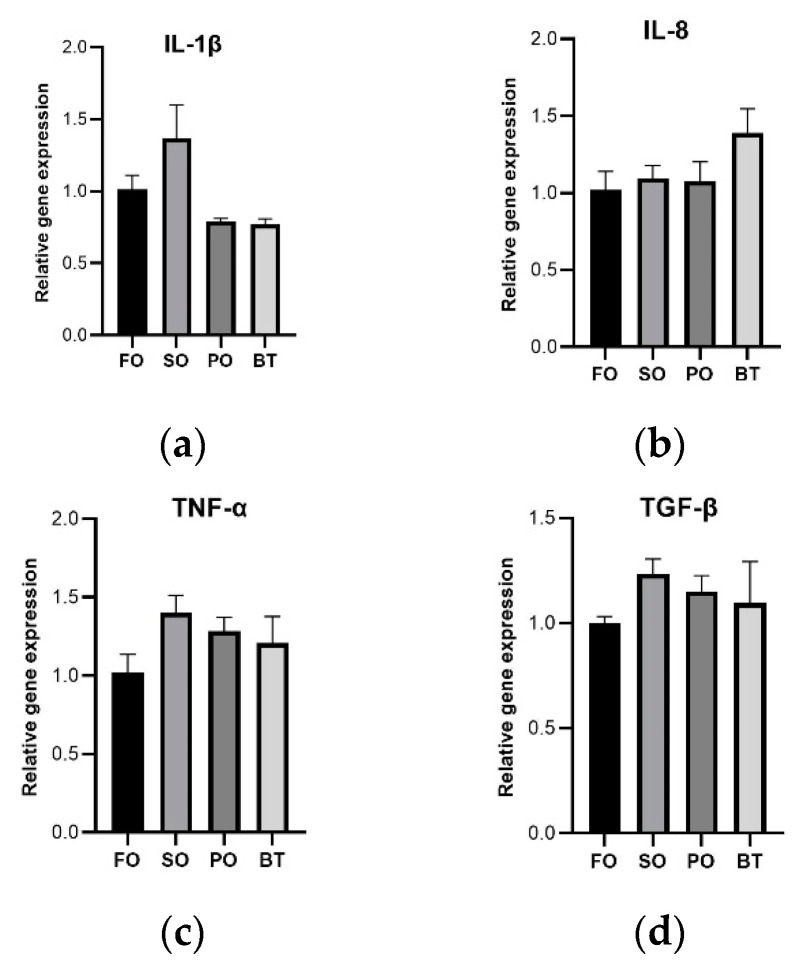
Effects of different diets on the expression of intestinal inflammatory factors of tiger puffer at the end of FOF period. (**a**) Expression of IL-1β; (**b**) Expression of IL-8; (**c**) Expression of TNF-α; (**d**) Expression of TGF-β. Values were means of triplicate tanks with standard errors. ^a, b^ For a certain parameter, value bars sharing a same superscript letter or those without superscript letter are not significantly different as evaluated by Tukey’s test (*p* < 0.05).

After FOF, there was no significant difference in expression of tight junction-related genes (Figure 5) and inflammation-related genes (Figure 6) among dietary groups.

### 3.4. Changes of Intestinal Microbiota Community Complexity under Fish Oil Finishing Strategy

**Figure 7:**
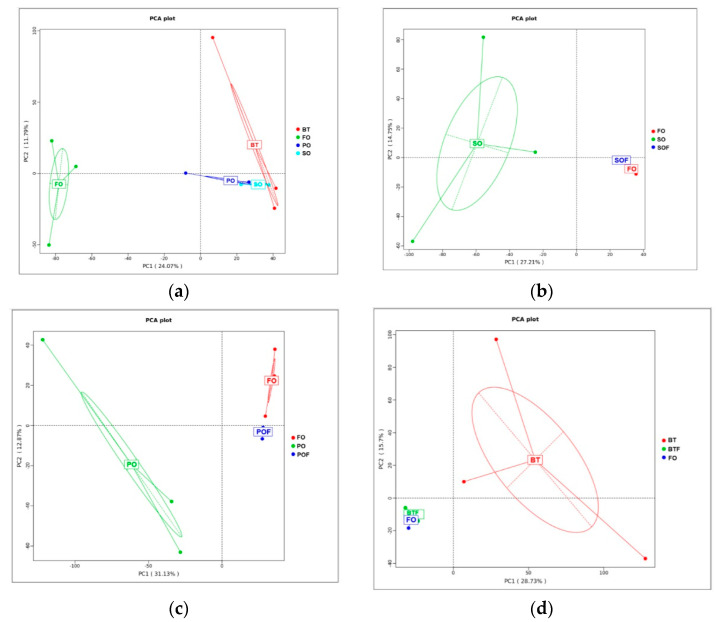
Beta−-diversity analysis of tiger puffer gut microbiota at the end of growing−out and FOF period. (**a**) Beta diversity of FO group and TSO group at the end of growing−out period; (**b**) Changes in β−diversity of the SO group in different periods. SOF represents the SO sample at the end of FOF period; (**c**) Changes in β−diversity of the PO group in different periods. POF represents the PO sample at the end of FOF period; (**d**) Changes in β−diversity of the BT group in different periods. BTF represents the BT sample at the end of FOF period.

The α diversity index includes Chao1 and Ace, which reflect species richness, and Shannon and Simpson index, which reflect species diversity. At the end of the growing-out period, the species richness of SO, PO and BT groups decreased significantly compared with the FO group (*p* < 0.05) (Table 3). The Shannon index of the SO, PO and BT groups also decreased significantly compared with the FO group (*p* < 0.05). After FOF, there was no significant difference in species richness and species diversity between TSO groups and the FO group. At the end of the growing-out period, the PCA analysis showed that the FO group and other treatment groups were obviously separately clustered (Figure 7a), while after FOF, the FO group and the TSO groups were concentrated in one cluster (Figure 7b–d).

### 3.5. Changes of Intestinal Microbiota Composition under Fish Oil Finishing Strategy

**Figure 8:**
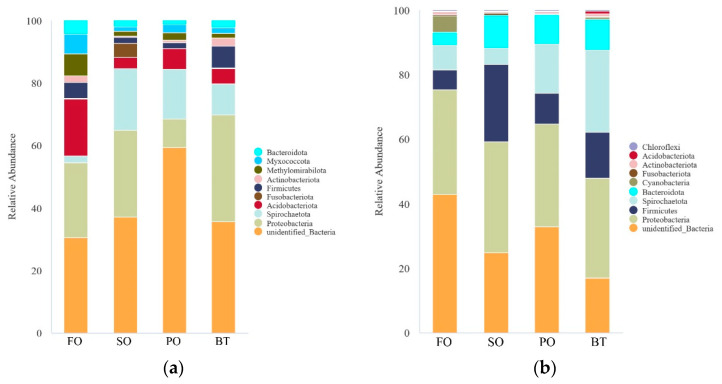
Effects of different diets on species abundance of tiger puffer gut microbiota composition at the phylum level at the end of growing-out (**a**) and FOF period (**b**).

At the phylum level, unidentified bacteria were not discussed. At the end of the growing-out period, the FO group was dominated by Proteobacteria and Acidobacteriota, while other groups were dominated by Proteobacteria and Spirochaetota. After FOF, the composition of each TSO group was similar to that of the FO group, and Proteobacteria was the dominant bacteria in most groups (Figure 8).

**Figure 9:**
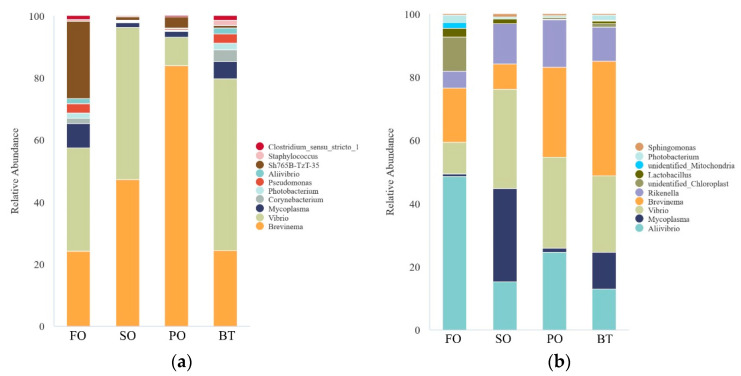
Effects of different diets on species abundance of tiger puffer gut microbiota composition at the genus level at the end of growing-out (**a**) and FOF period (**b**).

At the genus level, at the end of the growing-out period, the relative abundance of genera in the SO, PO and BT groups was significantly higher than that in the FO group. The dominant genera in the SO and BT groups were *Brevinema* and *Vibrio*, and the dominant genera in the PO group was *Brevinema*. After FOF, the distance in relative abundance between FO group and other TSO groups was shortened. The abundance of *Lactobacillus* in each TSO group increased significantly. The dominant genera of the SO group were *Vibrio* and *Mycoplasma*, and the dominant genera of the PO and BT group were *Brevinema* and *Vibrio*, respectively (Figure 9).

### 3.6. Specific Changes of Intestinal Microbiota in Response to FOF Strategy

**Figure 10:**
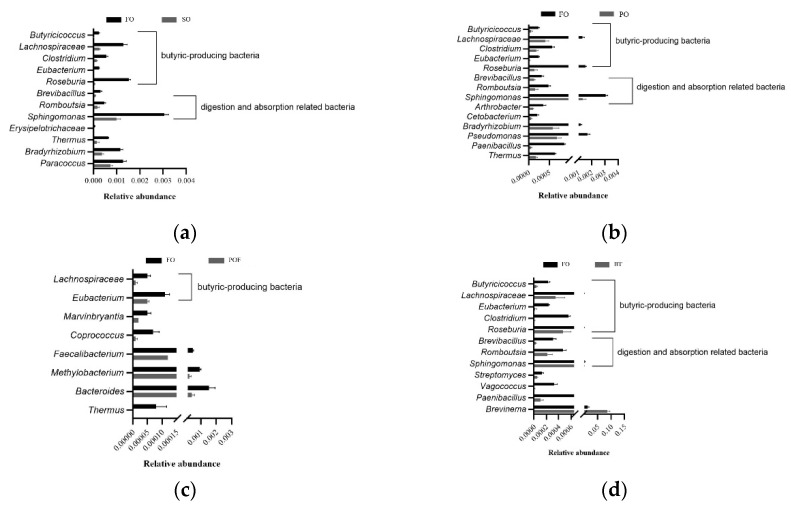
In the MetaStat analysis, the differential bacterial changes of different treatment groups at the end of growing-out and FOF period compared with the control group. (**a**) Changes of differential bacteria between SO group and FO group at the end of growing-out period; (**b**) Changes of differential bacteria between PO group and FO group at the end of growing-out period; (**c**) Changes of differential bacteria between POF group and FO group at the end of FOF period; (**d**) Changes of differential bacteria between BT group and FO group at the end of growing-out period.

In order to observe the specific changes in intestinal microbiota caused by FOF, two genus-level MetaStat analyses were performed. The results of first MetaStat analysis showed that the abundance of 12, 14 and 11 bacteria including *Sphingomonas* were significantly decreased in the SO, PO and BT groups, respectively, and that of *Brevinema* was significantly increased in the BT group. The second MetaStat analysis showed that the abundance of 12, 12 and 11 bacteria in the SO, PO and BT groups was significantly increased, respectively, during the FOF period (Figure 10).

## 4. Discussion

### 4.1. Effects of FOF on Nutrient Digestion and Absorption, Anti-Oxidative Capacity and Barrier Function of the Intestine

In this study, application of the FOF strategy for one month significantly restored the activities of digestive enzymes, antioxidant enzymes, and the integrity of the mucosal barrier. One of the reasons for the restorative effect of the FOF strategy on gut health may be due to the reintroduction of n-3 long chain polyunsaturated fatty acids (LC-PUFA), which is abundant in fish oil. n-3 LC-PUFA is not only more easily absorbed by fish and plays an important role in immune and inflammatory responses, it is also a key component of intestinal cell membranes, affecting the function and activity of transport proteins on the membrane [36,37,38,39].

These results suggested that the FOF strategy can recover the intestinal functional status and mucosal barrier weakened by feeding TSO. In addition, the growth results of this experiment, which have been published elsewhere, also confirmed the beneficial effects of FOF on fish growth [18]. These results could be also helpful to other pufferfish such as obscure puffer (*Takifugu rubripes*), which is a dominant aquaculture pufferfish species in freshwater but is supposed to have similar nutrient requirements [40,41,42].

### 4.2. Effect of FOF on Intestinal Microbiota

The α analysis showed that FOF for one month can significantly improve the intestinal microbiota richness and diversity reduced by feeding TSO. Usually, the species richness and diversity of the intestinal microbial community can reflect the ability of the community to resist the external environment, and the reduction of the richness and diversity can easily lead to the invasion of the intestinal tract by pathogens [43,44,45]. Therefore, the application of FOF strategy can significantly improve the stability of intestinal microbiota community of tiger puffer. This result was also evidenced by the PCA analysis. To assess whether the mitigating effects of FOF can lead to changes in ecological imbalance, the microbiota community distribution before and after FOF were compared. The results showed that the distance of each TSO group to the FO control group was shortened after FOF. By comparing the community before and after FOF, the intestinal microbiota community of tiger puffer experienced a process from disorder to recovery, which indicates that FOF for one month can alleviate the intestinal disorders by restoring the composition and complexity of intestinal microbiota.

The change of microflora abundance at the phylum level before and after FOF showed that the abundance of Proteobacteria increased significantly after FOF. Proteobacteria not only plays an important role in nitrification and anaerobic denitrification of marine sediments [46,47,48], but also widely exists in aquatic animals. It has even been reported that it has a 90.64% abundance dominance in the intestine of turbot [49]. In previous studies, Proteobacteria has also been confirmed to be dominant in the intestine of tiger puffer [50]. The abundance of Firmicutes also increased significantly after FOF. Many bacteria in this phylum, such as *Bacillus*, *Clostridium butyricum* and *Lactococcus*, have been widely reported in aquaculture as probiotics [51,52,53,54,55,56,57]. In addition, in the PO and BT groups, the ratio of Firmicutes/Bacteroides decreased after FOF. The lower F/B ratio is often considered as a healthier organism indicator [58,59].

At the genus level, the diversity of species composition increased after FOF. The abundance of *Brevinema* and *Vibrio*, which were originally dominant genera, decreased significantly after FOF. In general, *Brevinema* and *Vibrio* have been discussed as pathogenic bacteria in aquaculture. *Brevinema* was originally an infectious pathogen isolated from short-tailed shrews (*Blarina brevicauda*) and white-footed mice (*Peromyscus leucopus*) [60]. This bacterium was also found in the gills and intestines of rainbow trout and in the intestinal contents of Senegalese sole (*Solea senegalensis*) [61,62]. In the diseased seahorse (*Hippocampus kuda*), this bacterium was determined as a conditional pathogen, and its high abundance was closely related to the occurrence of intestinal diseases [63]. *Vibrio* is widely distributed in nature environments, especially in water. *Vibrio harveyi* is recognized as a serious pathogen in aquaculture [64], and diseased fish will suffer from gastrointestinal diseases, eye blindness, skin ulceration and other damages [65,66]. In shrimp culture, vibriosis is known as a major challenge, which easily causes liver and pancreas necrosis, as well as jejunum and death of shrimp [67,68,69]. In addition, the abundance of *Lactobacillus* increased significantly after FOF. As a common probiotic in aquaculture, *Lactobacillus* has been widely reported in various studies, including those on marine fish [70,71,72]. As a beneficial bacterium in the intestine, *Lactobacillus* can not only produce antibacterial substances and enhance intestinal immunity, but also improve the disease resistance of fish and promote the absorption and utilization of non-digestible carbohydrates [73,74,75,76].

In order to explore the specific changes of intestinal microbiota in response to FOF, two MetaStat analyses were analyzed in this study. The first MetaStat analysis compared the differential bacteria between the TSO groups and the FO group at the end of growing-out period. To some extent, these bacteria can be understood as significant changes caused by complete replacement of FO by TSO. The second MetaStat analysis compared the differential bacteria between the TSO groups and the FO group at the end of the FOF period. The results showed that some bacteria only appeared in the first MetaStat analysis. These bacteria were affected by the complete replacement FO by TSO, but can immediately recover to the same level of the control group after FOF. Two types of these bacteria were significantly regulated by FOF. The first type of bacteria was mainly butyric acid-producing bacteria, among which *Lachnospiraceae* and *Eubacterium* have obvious changes in the SO and BT groups, while *Butyricicoccus*, *Clostridium* and *Roseburia* have obvious changes in all the three TSO groups. *Lachnospiraceae* is a superfamily that produces butyrate, which can also decompose a variety of complex and indigestible carbohydrates for the host by degrading the main chain of cell wall polysaccharides [77,78,79]. Members of *Eubacterium* are involved in the degradation and fermentation of polysaccharides and promote the production of butyric acid [80,81,82]. *Butyricicoccus* can produce butyrate, which is essential to resist certain pathogens and maintain homeostasis [83]. In addition, some members of *Butyricicoccus* are closely related to the maintenance of the integrity of the intestinal mucosa [84]. *Clostridium* has been studied as probiotics for a long time [85]. *C. butyricum* under this genus is a butyric acid-producing bacteria, which can promote the absorption and utilization of nutrients and increase the weight gain rate of pacific white shrimp (*Litopenaeus vannamei*) [86,87]. As a probiotic in aquaculture, *Roseburia* also can decompose and utilize carbohydrates to produce propionic acid and butyric acid [88,89]. Butyric acid, as an important short-chain fatty acid, is the main energy source of intestinal epithelial cells, and has many physiological functions such as regulating intestinal flora and maintaining body fluid and electrolyte balance [90,91,92]. More importantly, butyric acid can inhibit the release of inflammatory factors, protect the intestinal mucosal barrier, reduce the inflammatory response, and maintain the intestinal immune balance [93,94,95]. Therefore, FOF can significantly increase the relative abundance of butyric acid-producing bacteria, which can improve the yield of butyric acid to a certain extent. It can be speculated that the positive effects of FOF on intestinal health may partly be mediated by the butyric acid regulation in the intestine.

The second type of bacteria that were obviously regulated by FOF were those related to digestion and absorption. After FOF, *Sphingomonas*, *Romboutsia* and *Brevibacillus* changed significantly in the SO, PO and BT groups. *Sphingomonas* not only can inhibit *Vibrio* to some extent, but also is a producer of cellulase, protease and amylase [96]. *Romboutsia* has been shown to be able to absorb and utilize carbohydrate, ferment amino acids, relieve inflammation, and maintain host health [97,98]. Studies have confirmed that *Brevibacillus* has the function of degrading cellulose and can degrade carboxymethyl-cellulose, cellobiose and microcrystalline cellulose [99]. Intestinal tract is the main place for nutrients digestion and absorption [100]. Studies also showed that microorganisms can be controlled through the signal of intestinal endocrine system and produce local effect to fatty acid transport in intestinal cells [101]. Therefore, the FOF strategy may also improve intestinal health by regulating the nutrient digestion and absorption and promoting the nutrients utilization.

## 5. Conclusions

The intestinal function and health of tiger puffer could be obviously recovered by the fish oil finishing (FOF) strategy. The composition and complexity of intestinal microbiota community were clearly regulated by the FOF strategy. FOF also exerts positive effects on butyric acid-producing bacteria and bacteria related to nutrient digestion and absorption. The present results shed novel insight into the response of fish intestinal micro-ecology to diet shift, and provide more comprehensive information when the FOF strategy is applied in fish farming practice.

## Figures and Tables

**Table 1:** Formulation and proximate composition of the experimental diets (% dry matter basis).

Ingredients	FO	SO	PO	BT
Fish meal	40.00	40.00	40.00	40.00
Soybean meal	16.00	16.00	16.00	16.00
Soybean protein concentrate	4.00	4.00	4.00	4.00
Wheat meal	21.48	21.48	21.48	21.48
Beer yeast	5.00	5.00	5.00	5.00
Casein	4.00	4.00	4.00	4.00
Mineral premix ^1^	0.50	0.50	0.50	0.50
Vitamin premix ^1^	0.20	0.20	0.20	0.20
Monocalcium phosphate	1.00	1.00	1.00	1.00
Vitamin C	0.20	0.20	0.20	0.20
Choline chloride	0.20	0.20	0.20	0.20
Attractant	0.30	0.30	0.30	0.30
Ethoxyquin	0.02	0.02	0.02	0.02
Mold inhibitor	0.10	0.10	0.10	0.10
Fish oil	6.00			
Soybean oil		6.00		
Palm oil			6.00	
Beef tallow				6.00
Lecithin	1.00	1.00	1.00	1.00
Proximate composition				
Crude protein	51.39	50.67	50.60	50.80
Crude lipid	10.09	10.37	10.37	9.94
Ash	10.13	10.19	10.24	10.19

^1^ Vitamin premix and mineral premix were the same as those used in previous study [28].

**Table 2:** Primer sequence and annealing temperature for the genes profiled in qPCR.

Gene	GenBank Accession no.	Sequences of Primers (5′→3′)	Annealing Temp./°C
IL-1β	NM_001280090.1	F: CATCACCCGCTGACCATGAA R: CATCCCTGAACTCGGTGCTC	62
TNF-α	AB183465.1	F: CTACTGGAACGGAAGGCAAGAGATG R: GATGCGGCTCAGCGTGTAGTG	60
TGF-β	NM_001280047.1	F: GCTCGATACCTCACTACTCGCTTAATC R: TCACAGAACAGCCGAAGTTGGAAG	61
IL-8	XM_035638413	F: GGCAGACCCCTTGAAGAATA R: TGGTGAACCCTTCCCATTAT	61
Claudin4	DR441341.1	F: TATTCTTTGGTGCTGCTCGGT R: TCTTGGGCGGAGCAATGTTA	62
Claudin7	AY554347.1	F: ATTCTTTGGTGCTGCTCGGT R: AGCAATGTTAGCTGCGTCCT	60
Claudin18	KU238180.1	F: CCAACTGCATTGATGACGAG R: GACCCCGGATACGATGAAGA	60
JAM-A	XM_003971244.3	F: CAAAAACGGCGTGCCTCTAC R: CCGAGTCCGACCTTGATGTT	60
ZO-1	XM_011610300.2	F: AGAGGTTACCCAAGGCCAGT R: CGTCCTGTCCCAGGAACAAA	62
β-actin	XM_003964421.2	F: ATCGTGCGTGACATCAAGGAGAAG R: TGTCCGTCAGGCAGCTCGTAG	61

**Table 3:** Changes of α diversity of intestinal microflora in tiger puffer.

	Observed Species	Chao1	Ace	Shannon	Simpson
**At the end of growing-out period**					
FO	3527.33 ± 58.15 ^b^	3632.64 ± 48.98 ^c^	3799.79 ± 45.96 ^c^	9.90 ± 0.06 ^b^	1.00 ± 0.00 ^b^
SO	2029.67 ± 190.90 ^a^	2124.00 ± 197.37 ^a^	2248.28 ± 203.65 ^a^	4.53 ± 0.84 ^a^	0.68 ± 0.15 ^ab^
PO	2452.67 ± 264.34 ^a^	2562.03 ± 273.40 ^ab^	2719.52 ± 286.41 ^ab^	5.30 ± 0.93 ^a^	0.69 ± 0.09 ^ab^
BT	2440.33 ± 289.41 ^a^	2546.87 ± 294.83 ^ab^	2700.53 ± 300.78 ^ab^	5.93 ± 1.24 ^a^	0.80 ± 0.12 ^ab^
	**At the end of FOF period**				
FO	1548.67 ± 91.73 ^abc^	1977.43 ± 94.57	2245.61 ± 92.17	3.52 ± 0.50	0.65 ± 0.12
SO	1320.33 ± 32.64 ^abc^	2145.95 ± 41.19	2400.51 ± 57.07	3.68 ± 0.24	0.78 ± 0.03
PO	1254.33 ± 26.19 ^ab^	2020.56 ± 81.32	2212.14 ± 128.38	3.50 ± 0.23	0.80 ± 0.04
BT	1673.33 ± 188.18 ^bc^	2415.78 ± 261.90	2611.84 ± 312.01	4.29 ± 0.20	0.85 ± 0.02

## Data Availability

The data that support the findings of this study are available from the corresponding author upon reasonable request.
